# Synthesis, Characterization and DNA-Binding Affinity of a New Zinc(II) Bis(5-methoxy-indol-3-yl)propane-1,3-dione Complex

**DOI:** 10.3390/ph14080760

**Published:** 2021-08-03

**Authors:** Luca Scapinello, Guglielmo Vesco, Luca Nardo, Angelo Maspero, Federico Vavassori, Simona Galli, Andrea Penoni

**Affiliations:** Department of Science and High Technology, Università degli Studi dell’Insubria, Via Valleggio 9, 22100 Como, Italy; luca.scapinello@uninsubria.it (L.S.); gvesco@uninsubria.it (G.V.); luca.nardo@uninsubria.it (L.N.); f.vavassori1@uninsubria.it (F.V.); simona.galli@uninsubria.it (S.G.); andrea.penoni@uninsubria.it (A.P.)

**Keywords:** zinc(II) complexes, indoles, β-diketone, DNA ligand, metallodrug, DNA-targeted chemotherapy

## Abstract

The novel zinc(II) µ-oxo-bridged-dimeric complex [Zn_2_(µ-O)_2_(BMIP)_2_] (BMIP = 1,3-bis(5-methoxy-1-methyl-1*H*-indol-3-yl)propane-1,3-dione), **1**, was synthetized and fully characterized. The spectral data indicate a zincoxane molecular structure, with the BMIP ligand coordinating in its neutral form via its oxygen atoms. Structural changes in **1** in dimethylsulfoxide (DMSO) were evidenced by means of spectroscopic techniques including infrared absorption and nuclear magnetic resonance, showing DMSO entrance in the coordination sphere of the metal ion. The resulting complex [Zn_2_(µ-O)_2_(BMIP)_2_(DMSO)], **2**, readily reacts in the presence of *N*-methyl-imidazole (NMI), a liquid-phase nucleoside mimic, to form [Zn_2_(µ-O)_2_(BMIP)_2_(NMI)], **3**, through DMSO displacement. The three complexes show high thermal stability, demonstrating that **1** has high affinity for hard nucleophiles. Finally, with the aim of probing the suitability of this system as model scaffold for new potential anticancer metallodrugs, the interactions of **1** with calf thymus DNA were investigated in vitro in pseudo-physiological environment through UV-Vis absorption and fluorescence emission spectroscopy, as well as time-resolved fluorescence studies. The latter analyses revealed that [Zn_2_(µ-O)_2_(BMIP)_2_(DMSO)] binds to DNA with high affinity upon DMSO displacement, opening new perspectives for the development of optimized drug substances.

## 1. Introduction

Since the anticancer properties of cisplatin were first discovered by Rosenberg in 1965 [1], many efforts have been focused on both finding more effective metallodrugs and reducing the severe side effects typical of the parent compound [2,3,4,5]. In particular, β-diketone-based metal complexes are constantly catching interest [6,7] due to β-dicarbonyls’ structural simplicity and, in case of, e.g., curcuminoid compounds, intrinsic biological properties and natural availability [8]. For instance, Lippard reported benzoylacetonate platinum(II) complexes to preserve antitumor activity, in some cases even at lower doses than uncomplexed cisplatin [9]. Among non-platinum based compounds, in 1995, Hanauske and coworkers introduced budotitane as the first titanium(IV) drug for cancer treatment [10]. Other cytotoxic β-dicarbonyls exploit the presence of Ru^II^ [11], Co^II^ [12], Cu^II^ [13,14], Ni^II^ [15,16] and oxovanadium(IV) [17].

Among cheap and low-toxicity metal ions of the d-block, zinc(II) is one of the most abundant ones naturally present in the human body [18,19] and plays key roles in cell metabolism [20,21]. Recently, many Zn^II^ complexes with good DNA-binding properties were synthetized, exploiting different types of bidentate ligands such as bis(thiosemicarbazone) [22], isoeuxanthone [23], 5,7-dihalo-8-substituted quinolones [24], oxolinic acid [25] and Hpyramol [26]. However, only few examples of cytotoxic Zn^II^ β-diketone-based metallodrugs are reported in the literature, containing either acetylacetone [27], 2-thenoyltrifluoroacetone [28] or curcuminoid fragments [29,30,31]. 

**Scheme 1:**
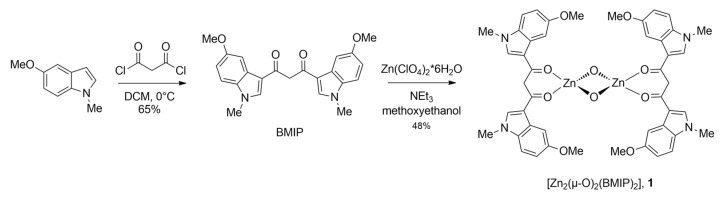
Synthesis of BMIP and [Zn_2_(µ-O)_2_(BMIP)_2_], **1**.

Indole derivatives have tremendous relevance for their biological importance [32,33,34] and they are subjected to extensive research for their anticancer properties [35,36,37]. In 2010, Cirrincione and coworkers provided new bis(indol-3-yl)propane-1,3-dione molecules as intermediates to achieve nortopsentin analogues with antitumor potential [38]. Given our general interest in indole chemistry [39,40,41] and inspired by Cirrincione’s work, we present in this paper the synthesis and characterization of a novel Zn^II^ complex, [Zn_2_(µ-O)_2_(BMIP)_2_], **1**, (Scheme 1). A deep analysis of the molecular structure of **1** has been carried out, revealing a zincoxane core which binds the BMIP ligand in neutral form. Compound **1** shows good affinity for hard nucleophiles, as it can readily expand its coordination sphere when dissolved in DMSO, forming the [Zn_2_(µ-O)_2_(BMIP)_2_(DMSO)] species, **2**. Moreover, the DMSO ligand can be easily displaced by *N*-methyl-imidazole (NMI), a common nucleoside mimic, leading to the formation of [Zn_2_(µ-O)_2_(BMIP)_2_(NMI)], **3**. The three compounds show high thermal stability. Finally, successful DNA coordination tests in pseudo-physiological buffer solution suggest that **1** can be regarded as a scaffold compound for a new family of potential anticancer drugs.

## 2. Results and Discussion

### 2.1. Synthesis

Synthesis of the BMIP ligand was accomplished by acylation of the electron-rich 5-methoxy-*N*-methyl-indole with malonyl dichloride [38]. Compound **1** was afforded by reaction of BMIP with zinc(II) perchlorate in basic conditions (Scheme 1). Further details are given in the Supplementary Materials section.

### 2.2. Spectroscopic and Structural Studies

#### 2.2.1. NMR Spectroscopy

**Scheme 2:**
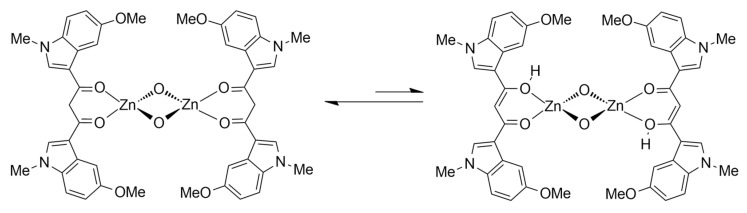
Keto-enol tautomerism in **1**.

As the ^1^H-NMR spectrum of **1** in CD_2_Cl_2_ shows a symmetrical aromatic pattern (see Supplementary Materials Figure S1), BMIP metal chelation via the carbonyl oxygens can be assumed. Concerning the upfield region, the signals for the indole *N*-Me and the 5-methoxy groups were found overlapped at 3.76 ppm, whilst in free BMIP they are observed as distinct singlets at 3.75 and 3.77 ppm, respectively. More interestingly, the ^1^H-NMR spectrum of **1** shows a signal at 4.32 ppm, similar to that found for the β-dicarbonyl CH_2_ in the free ligand, the associated integral being compatible with two protons. This suggests that BMIP mainly coordinates via its neutral keto form, rather than its enolate (Scheme 2). This is supported also by the ^13^C-NMR spectrum (Supplementary Materials Figure S2), clearly evidencing not only the β-dicarbonyl CH_2_ at 55 ppm, but even the ketone carbon signal at 188 ppm. Many examples of chelating neutral diketone complexes are already documented in the literature, mainly containing acetylacetone [42,43,44]. However, if BMIP is present as neutral ligand, an anionic charge compensator for Zn^II^ must be present. Lewinski recently reported that a characteristic zincoxane Zn-O-Zn bridged structure is quite common in zinc(II) coordination chemistry [45]. In particular, treatment of zinc(II) acetylacetonate in refluxing polar solvents leads to formation of Zn-O clusters [46].

**Figure 1:**
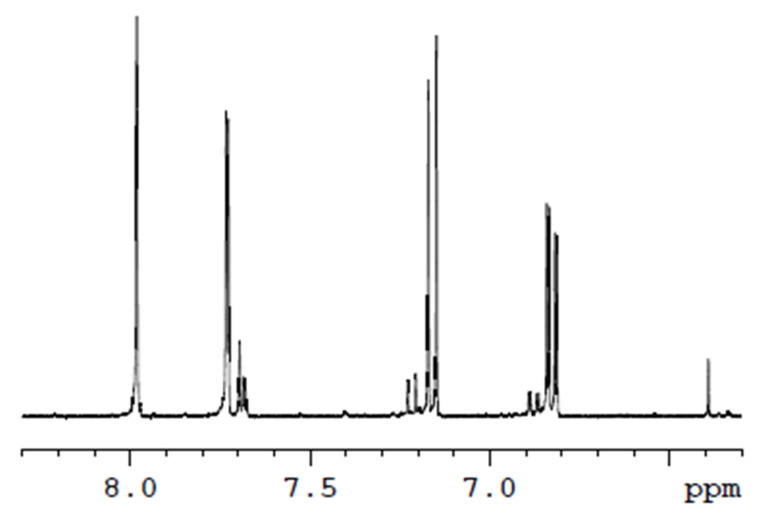
Zoom of the ^1^H-NMR spectrum of **1** on the signals used to quantify the fraction of diketo tautomer.

The stoichiometry of complex **1**, determined by assessing its molecular weight with high-resolution mass spectrometry (HRMS) and elemental analysis, points to the formation of a Zn^II^ dinuclear complex, formally viewed as two [Zn(BMIP)]^2+^ units with the zinc(II) centers bridged by two µ_2_-O^2−^ anions (Scheme 1). The presence of µ_2_-O^2−^ is required to neutralize the positive charge of the [Zn(BMIP)]^2+^ unit. The molecular structure of **1** may then be formally considered as a dimeric zincoxane stabilized by the organic ligand. In addition, the presence in the ^1^H-NMR spectrum of **1** of side peaks in the aromatic region at 7.70 ppm, 7.68 ppm, 7.22 ppm and 6.88 ppm, and of the characteristic olefin proton at 6.50 ppm (see Figure 1) reveals the coexistence of the enolic neutral form (Scheme 2) together with the prevalent diketo one. The presence of the enol tautomer in neutral coordinated β-dicarbonyls is already documented in the literature [42,43].

#### 2.2.2. IR Spectroscopy

The FT-IR analysis of compound **1** (Table 1 and Supplementary Materials Figure S3) reveals that the C=O stretching band is located at 1520 cm^−1^, strongly red-shifted compared to the homologous vibration at 1618 cm^−1^ detected for the BMIP free ligand. This marked difference is a good evidence of strong coordination of the metal ion through the β-diketone oxygen atoms [47]. Furthermore, two additional bands lacking in the ligand spectrum appear in the low frequency region at 507 cm^−1^, which may be attributed to Zn-O_BMIP_ vibrations [48].

#### 2.2.3. Electronic-State Transition Spectroscopy

##### UV-Vis Absorption in Organic Solvents

**Figure 2:**
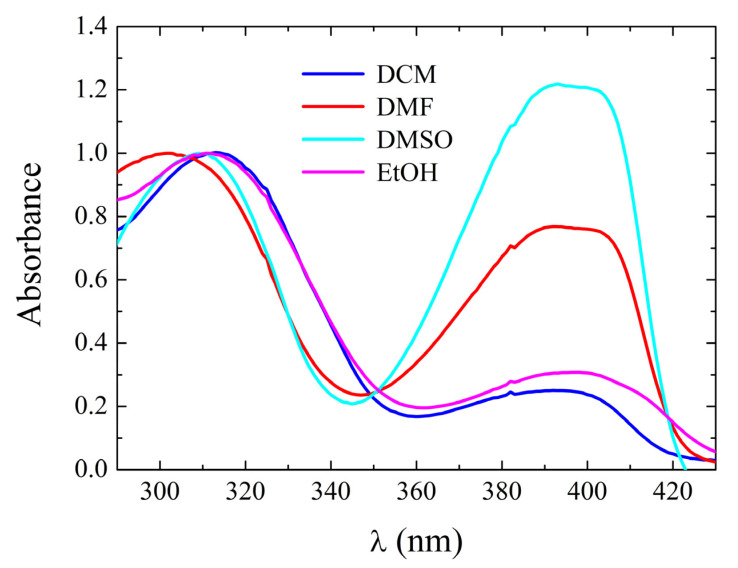
Absorption spectra of **1** in selected solvents.

In order to further investigate the keto-enol tautomerism of **1** in different environments, UV-Vis absorption spectroscopy experiments were undertaken. Namely, the UV-Vis absorption spectra of **1** were recorded in a panel of organic solvents and in water. Unfortunately, due to the low solubility of **1** in most environments, only a limited number of solvents could be probed, namely: dichloromethane (DCM), as a benchmark of slightly polar poorly hydrogen-bonding solvent; DMSO and dimethylformamide (DMF), as benchmarks of highly polar, H-bond accepting solvent; and ethanol (EtOH), as a paradigm of protic solvent. In EtOH, **1** is only slightly soluble (saturation is reached in the micromolar concentration range). Thus, filtration of the solution over paper was necessary to obtain clear (i.e.,: non-scattering) samples. The absorption spectra are plotted in Figure 2. Due to the high absorption of DMSO and DMF in the UVB spectral region, our analysis has focused on the 270–650 nm range. The spectra acquired in any of the tested solvents exhibit two distinct absorption peaks, one around λ_abs,1_ ≈ 310 nm and another at λ_abs,2_ ≈ 395 nm. The exact positions of the absorption peaks are reported in Table 2.

The peaks positions and bands’ line-shapes are only slightly affected by the solvent properties. Conversely, the relative intensity of the two bands dramatically changes, as seen in Table 2, fourth column, where the values of f_1_ = Abs(310 nm)/[Abs(310 nm) + Abs(395 nm)] are reported.

Relying on previous experiences [49,50] as well as literature data [51] on other β-diketones, we attributed the 310 nm peaked band to the absorption of the BMIP ligands in the diketo tautomer, and the 395 nm peaked one to the absorption of those in the enol form. According to this interpretation, the coexistence of two peaks in all the solvents would support the picture offered by NMR, suggesting that both the diketo and the enol tautomers are able to chelate zinc(II) ions with comparable efficacy, and that the keto-enolic equilibrium is primarily dictated by solute–solvent interactions, rather than complexation.

The bands’ attribution is corroborated by comparison of the relative peak absorbance f_1_, retrieved from the UV-Vis spectra, with the relative integral of the signals at 7.16 ppm and 7.22 ppm, characteristic of the diketo and enol tautomers, respectively, in the ^1^H-NMR spectra in deuterated DCM and DMSO (see fifth column of Table 2). Unfortunately, the acquisition of NMR spectra in EtOH was prevented by the scarce solubility of **1** in this solvent, while we did not investigate the equilibrium in deuterated DMF due to shortage of this solvent. However, the almost perfect matching of the relative absorbance with the fractional concentration values in both the solvents used for double-blind analysis by means of UV-Vis and NMR spectroscopy (Table 2, columns 4 and 5) implies that, at least in DCM and DMSO, the molar extinction coefficient is very similar at the diketo and keto-enol absorption peak. Indeed, estimation of this spectroscopic parameter is feasible on solutions in which the total concentration of **1** is predetermined by weight (see Materials and Methods for details). In such specimens, the fractional concentrations of diketo and enol tautomers established by NMR can be adopted to calculate the absolute diketo and enol concentration values. The resulting molar extinction coefficients (reported in parenthesis beneath the peak wavelengths of the pertaining absorption peaks in the second and third column of Table 2) differ by few percent. Under the assumption that this similarity holds true also in the other solvents, the relative absorbance values reported in the fourth column of Table 2 constitute good estimates of the fractional concentration of diketo tautomer also in DMF, and the average molar extinction coefficient can be calculated (see last column in Table 2). Due to the necessity of preliminarily filtering the EtOH solution, it was impossible to derive the molar extinction coefficient value in this solvent, instead.

##### Steady-State Fluorescence in Organic Solvents

**Figure 3:**
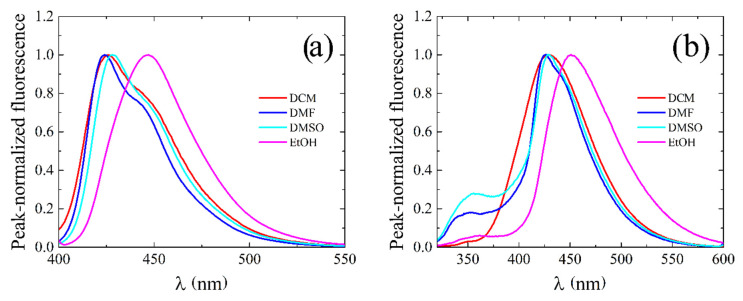
Fluorescence emission spectral line-shapes of **1** in different solvents upon excitation at: (**a**) the enol, and (**b**) the diketo absorption peak.

The fluorescence emission spectra of **1** in the above-investigated solvents were recorded upon excitation at both the enol and the diketo absorption peak. They are plotted in Figure 3, panels (a) and (b), respectively. Excitation at the ≈395 nm (enol) peak results in an emission peaked at around 425 nm, with the exception of the notably red-shifted spectrum measured in EtOH (λ_max_ ≈ 450 nm). The exact values of the maximum emission wavelength in the different solvents are listed in Table 3.

A similar behavior (red-shifted fluorescence in alcohols) was observed for several curcuminoids, and ascribed to tautomerism between the open *cis-* and *trans-*enol species [49,50,52,53,54,55,56,57]. The quantum yield of **1** is generally low (see the values reported in Table 3). In spite of this, excitation at the ≈310 nm (diketo) peak results in spectra dominated by out-of-peak excited emission of the enol tautomers, while the emission of the diketo ones builds up a secondary band peaked at ≈350 nm, suggesting an even lower quantum yield for the latter tautomers.

This attribution is corroborated by the fluorescence excitation spectra recorded with λ_obs_ = 425 nm and 350 nm, which peak at ≈395 nm and around 300 nm, respectively (see Supplementary Materials Figure S4). The only exceptions to this rule are the excitation spectrum recorded with λ_obs_ = 425 nm in DCM, which has a maximum at 326 nm due to the high prevalence of the diketo tautomers in this solvent, and the excitation spectrum with λ_obs_ = 350 nm in DMF, which peaks at 280 nm, with a marked shoulder above 300 nm. Again by analogy with previously reported studies on curcumin analogues [49,50], this might indicate a non-trivial *cis-*/*trans-*tautomerism between the diketo tautomers in this solvent. Subtraction of out-of-peak excited fluorescence by the enolic species allowed to reconstruct the spectral features of the diketo emission (see Supplementary Materials Figure S5).

##### Spectroscopic Properties in Aqueous Environment

**Figure 4:**
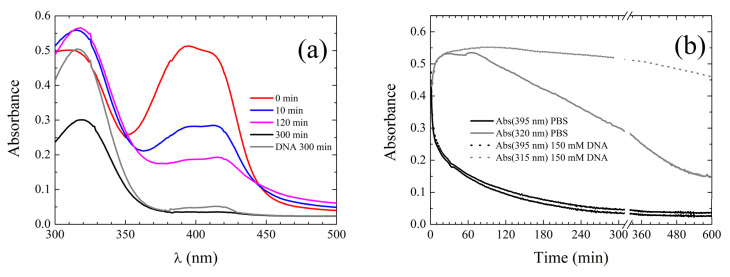
(**a**) Selected absorption spectra during a time-lapse experiment aimed at monitoring hydration dynamics of **1** preliminarily dissolved in DMSO; (**b**) absorbance at selected wavelengths versus time in the same experiment. See text for details.

With the final goal to characterize the binding affinity of **1** for DNA in pseudo-physiological environment, a spectroscopic characterization of **1** in aqueous solution (namely phosphate buffer at pH 7.4 and 10 mM ionic strength) was undertaken. These investigations were hampered by the very low solubility of **1** in water. The low ionic strength adopted was indeed necessary in order to prevent ready precipitation of the compound. Nonetheless, in order to attain a clear solution, preliminary solubilization in DMSO followed by re-equilibration in buffer was necessary. In Figure 4a, selected spectra are plotted of a time-lapse experiment in which 50 μL of a 880 μM concentrated stock of **1** in DMSO are added to 2.95 mL of buffer. Readily after addition, the absorption spectrum (red curve) is very similar to that detected for a solution of **1** in pure DMSO (see Figure 2 for comparison). However, as solvent rearrangement takes place, DMSO is removed from the microenvironment of the compound and the latter is hydrated, the absorption peak at 390 nm characteristic of the enol tautomers progressively decreases in intensity at the advantage of a peak at 320 nm, which we ascribe to the absorption of hydrated diketo tautomers. Concomitantly, the peak at 310 nm typical of the diketo tautomers in DMSO also reduces its intensity. As shown in Figure 4, panel (b), where the absorbance values at 320 nm and 393 nm are plotted as a function of time, within 5 h after addition of the stock, the spectrum, plotted in black in Figure 4a, appears constituted by a single band peaked at 320 nm. This observation agrees with previous reports on the superior water solubility of the diketo with respect to the enol tautomers of synthetic curcuminoids [51]. Nonetheless, at length even the diketo tautomer tends to aggregate and precipitate, as denounced by progressive reduction in the absorbance at 320 nm.

**Figure 5:**
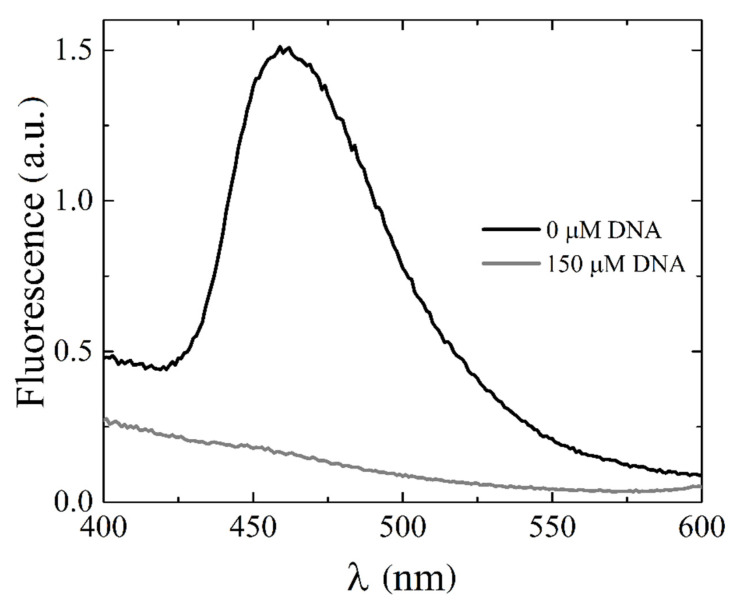
Fluorescence emission spectra of **1** in aqueous environment in the absence (black line) and presence (gray line) of DNA. The compound was preliminarily solubilized in DMSO. The samples were excited at 320 nm.

For this reason, during the measurements in aqueous environment reported hereafter, where not otherwise stated, the samples were kept under stirring. Upon excitation in correspondence to the 320 nm peak of solutions kept under stirring overnight to achieve completion of solvent rearrangement, a fluorescence peak at 460 nm was detected (see Figure 5 for the emission spectrum). The quantum yield was measured to be 0.02.

##### Binding to DNA

The same measurements performed in aqueous environment were repeated upon addition of 50 μL of the same stock of **1** in DMSO to 2.95 mL of buffer in which 150 μM calf thymus DNA had been preliminarily dissolved. The solvent rearrangement dynamics resulted to be minimally affected by the presence of DNA. However, after complete hydration, the aggregation/precipitation dynamics take place with significantly slower kinetics (see Figure 4b, dotted lines). We interpret this phenomenon as due to binding of **1** to DNA and consequent slower precipitation of the complex.

The absorption spectral line-shape is slightly blue shifted to ≈315 nm. However, a sizeable hyperchromicity is observed, as shown in Figure 4a (gray line). Moreover, a very faint band appears peaking around 400 nm, i.e., in the absorption region typical of the enol tautomers. A similar behavior also pertains to aqueous solutions with and without DNA in which **1** is added upon preliminary solubilization in DMF, as shown in Supplementary Materials Figure S6a. In the latter case, absorption in the enol band is not detected.

The most striking effect of DNA addition concerns fluorescence emission. Namely, the fluorescence intensity is reduced to a barely detectable signal, see Figure 5, gray line, and Figure S6b, for the spectrum detected on the samples added with **1** dissolved in DMSO and DMF, respectively.

**Figure 6:**
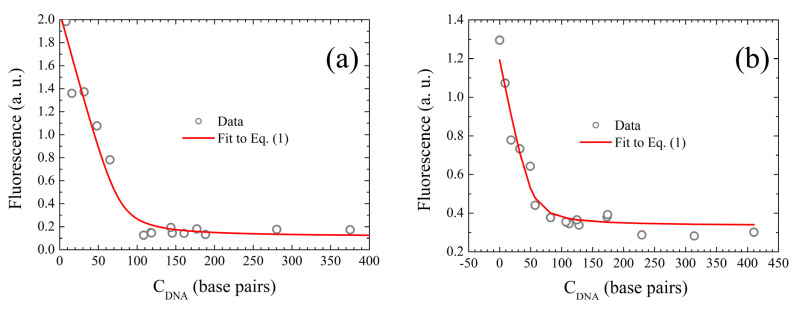
Binding isotherms of the binding of **1** to calf thymus DNA in 10 mM phosphate buffer. In order to dissolve the ligand in water, equal aliquots of concentrated stocks in organic solvents miscible with water were added to aqueous solutions of DNA in different concentrations. In panel (**a**), the organic solvent was DMSO and the final ligand concentration was 20 μM; in panel (**b**), DMF was used and the ligand was added to a final concentration of 15 μM. In both the experiments, the volume-to-volume concentration of organic solvent in phosphate buffer was 1:150.

Accordingly, fluorescence quenching was assumed as a binding indicator and titration was attempted with the aim of estimating the apparent binding constant of the reaction. The details on samples preparation are provided in the Materials and Methods section. The binding isotherms (i.e., graphs of fluorescence at 460 nm plotted as a function of DNA concentration) obtained starting from **1** dissolved in DMSO and DMF are shown in Figure 6, together with the pertaining best-fitting curves according to the independent binding site model [58,59], i.e., using the following equation:

(1) $$F\left(C_{\mathrm{DNA}}\right)=F\left(0\right)+\frac{\delta F}{2}\left[{\mathrm{nK}}_{\mathrm{diss}}+D+C_{\mathrm{DNA}}-\sqrt{{\left({\mathrm{nK}}_{\mathrm{diss}}+D+C_{\mathrm{DNA}}\right)}^{2}-4{\mathrm{DC}}_{\mathrm{DNA}}}\right]$$

In Equation (1) F(C_DNA_) is the fluorescence measured for a solution at D concentration of **1** and C_DNA_ concentration of DNA base pairs. D was kept constant to the values of 20 μM and 15 μM for the titrations starting from the DMSO and DMF stocks, respectively, and fixed accordingly during the fit. F_0_ represents the fluorescence of the free dye, δFxD the difference in fluorescence of the dye when totally bound to DNA with respect to F_0_. The parameters descriptive of the binding affinity are the dissociation constant, K_diss_, and the number of molecules of **1** reacting per DNA base pair in conditions of full binding sites saturation, n (i.e., the inverse of the number of bases involved in the binding of a single ligand molecule, the so-called binding length). The detailed fitting parameters are reported in Table 4. The apparent affinity constant for the binding reaction, defined as n/K_diss_, resulted to be on the order of 10^6^ M^−1^ for both titrations. This value is comparable to those tabulated for several well established DNA stains and DNA-binding drugs [58]. The fairly low value retrieved for n, corresponding to a binding length of four base pairs, reasonably reflects the bulkiness of **1**.

#### 2.2.4. Powder X-ray Diffraction Studies

Powder X-ray diffraction qualitative analysis revealed the reproducibility of the synthetic path proposed for **1**, not only at the molecular level, but also in the solid state: indeed, the precipitate, recovered from the reaction medium in the form of microcrystalline powders, does show the same PXRD pattern (Figure S7). Unfortunately, attempts to retrieve the unit cell parameters as the first step to undertake a crystal structure determination from PXRD invariably failed, irrespective of the software used. Incidentally, due to the already mentioned low solubility of **1** in different organic solvents, all the efforts carried out to recrystallize it in the form of single crystals suitable for single-crystal X-ray diffraction were not successful.

### 2.3. Coordination with DMSO and NMI

Although many zinc(II) coordination compounds are stable in a tetrahedral geometry, some five-coordinated complexes have been reported in recent years [60,61,62]. One notable example is provided by DMSO coordination [63]. As **1** is optimally soluble in this organic solvent, we investigated the proneness of DMSO to act as extra ligand in the coordination sphere of **1**. Detailed synthetic procedures are given in the Supplementary Materials section.

**Figure 7:**
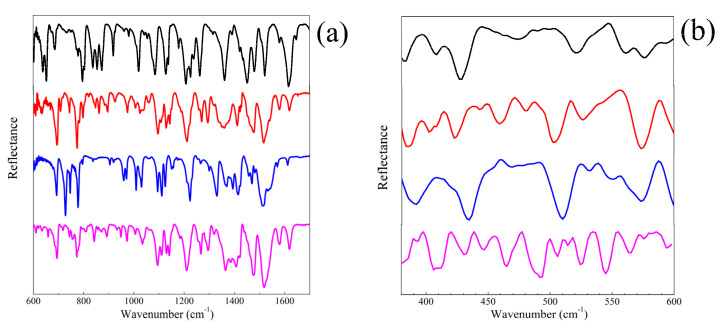
IR spectra of BMIP (black lines) **1** (red lines), **2** (blue lines), and **3** (magenta lines) in (**a**) the 600–1700 cm^−1^, and (**b**) 380–600 cm^−1^ spectral ranges.

DMSO coordination to form [Zn_2_(µ-O)_2_(BMIP)_2_(DMSO)], **2**, was indeed observed by solid state IR spectroscopy. The band positions are listed in Table 1. The DMSO diagnostic bands at 1031 (methyl rocking) and 961 cm^−1^ (S=O vibration) were identified, as shown in Figure 7a. The red-shifted position of the latter band (the S=O vibration for free DMSO falls at 1054 cm^−1^) suggests that DMSO binds to the metal center through the oxygen atom. This is further confirmed by the presence of a band at 435 cm^−1^, attributed to Zn-O_DMSO_ stretching [64], as shown in Figure 7b. BMIP coordination is minimally affected by DMSO binding. Indeed, its characteristic carbonyl band is observed at 1520 cm^−1^, not shifted with respect to the tetra-coordinated complex, while the low frequency peak at 507 cm^−1^ ascribable to Zn-O_BMIP_ stretching is only slightly blue-shifted to 510 cm^−1^.

**Scheme 3:**
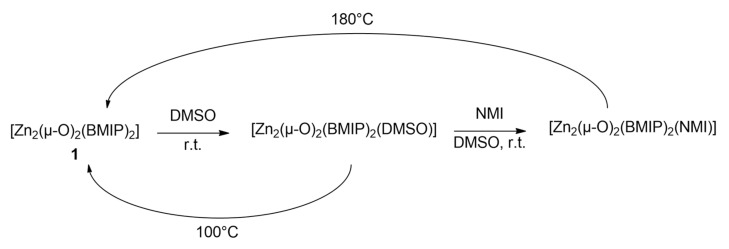
Reversible coordination of **1** with DMSO and NMI to yield **2** and **3**, respectively.

DMSO coordination is also unravelled by solution NMR. The DMSO-characteristic methyl signal at 2.58 ppm is identified in the ^1^H-NMR spectrum, along with an unmodified BMIP aromatic pattern and the tautomeric equilibrium, see Supplementary Materials Figure S8. The [Zn_2_(µ-O)_2_(BMIP)_2_(DMSO)] stoichiometry was confirmed by combined thermogravimetric analysis (TGA) and differential scanning calorimetry (DSC). The TGA profile shows that species **2** decomposes in two subsequent steps (see Supplementary Materials Figure S9). The first one, occurring at 184 °C, is compatible with the removal of the DMSO molecule (experimental mass loss: 7.92%, theoretical: 7.70%), and coincides with an endothermic event detected by means of DSC. The second step, which starts at 310 °C and ends up with a 37.78% mass loss, corresponds to decomposition of **1**. The DSC/TGA of **1** is shown in Supplementary Materials Figure S10. Indeed, after DMSO loss, the DSC and TGA curves of **2** follow the same trend observed for compound **1**. Although the high temperature required for DMSO ablation denotes the high stability of the DMSO complex, the similar trend of the DSC/TGA curves measured for **1** and **2** at high temperature suggests that DMSO ligation should be reversible. Accordingly, by heating **2** at 100 °C for 3 h under vacuum, **1** was reobtained in quantitative yield (Scheme 3), as testified by IR spectroscopy.

In conclusion, the above analyses demonstrate the ability of **1** to tune the zinc(II) coordination geometry in the presence of electron donors. This ability might provide a means for zinc(II) coordination with DNA nucleosides, as witnessed by the results exposed in Section 2.2.3. The next step towards characterization of the binding mode of **1** to DNA was to attempt coordination with *N*-methyl-imidazole (NMI), a widely used DNA mimic. Namely, **1** was initially suspended overnight under stirring in NMI, filtered, washed thoroughly to remove NMI residuals, and dissolved in CD_2_Cl_2_. Formation of [Zn_2_(µ-O)_2_(BMIP)_2_(NMI)], **3**, was evidenced by solution ^1^H-NMR, namely by the appearance of the diagnostic *N*-Me signal at 3.71 ppm, as shown in Supplementary Materials Figure S11. Coordinated NMI aromatic protons were spotted as broad signals at 7.56 ppm, 7.07 ppm and 6.95 ppm, notably downfielded compared to the free molecule. The experiment was repeated starting from **2**. In this instance, displacement of DMSO by NMI was observed. Indeed, the ^1^H-NMR spectrum was characterized by the disappearance of the signal at 2.58 ppm diagnostic of DMSO with concomitant appearance of the above-described features signalling NMI complexation. Finally, NMI complexation was afforded in CH_2_Cl_2_, adding the DNA mimic only in low amount (10 % *v/v*).

The infrared spectra of **3**, see Figure 7, show two intense absorption bands at 1285 cm^−1^ and 1421 cm^−1^, attributed to the C–N and C=N ring stretching vibrations of NMI, respectively. Furthermore, when **2** is used as the starting compound, the bands characteristic of DMSO complexation (1031 cm^−1^, 961 cm^−1^ and 435 cm^−1^, vide supra) disappear.

The thermal behaviour of **3** was investigated by simultaneous DSC/TGA. The TGA trace shows that **3** decomposes in two steps (Supplementary Materials Figure S12). The first one, occurring at ≈ 220 °C, corresponds to removal of one NMI mole per mole of complex (experimental mass loss: 8.14%, theoretical: 8.0%), in agreement with the endothermic broad peak located at 253 °C (DSC). The second step occurs in the >360 °C temperature range, with a mass loss of 43%, and corresponds to an endothermic decomposition process. After loss of NMI, the DSC/TGA curve of **3** is very similar to that of **1**.

Full coordination reversibility was also observed in this case, leading to quantitative recovery of compound **1** after 3 h of vacuum heating at 180 °C (Scheme 3).

## 3. Materials and Methods

### 3.1. Chemicals and Samples Preparation

All the chemicals were purchased from commercial suppliers and used as received. All reactions involving air- and/or moisture-sensitive materials were carried out under inert atmosphere, using a standard Schlenk-line technique. Where specified, reactions were monitored by thin layer chromatography (TLC), using ready-made ALUGRAM Xtra SIL G/UV254 (0.2 mm thick layer, Macherey-Nagel). Spots were observed under a UV lamp (365 nm and/or 254 nm). Gravimetric column chromatography was performed using silica gel as stationary phase (0.63–2 μm particle size).

The samples in aqueous environment exploited in the electronic-state transition spectroscopy characterization and in the spectrophotometric titration experiments were derived by highly concentrated stocks of **1** preliminarily solubilized in water-miscible organic solvents, namely DMSO and DMF. Indeed, due to the very scarce water solubility of **1,** attempts to obtain clear water solutions by directly pouring powdered **1** in buffer failed, in spite of heating, sonication or overnight stirring. First, the solvent rearrangement dynamics (i.e., progressive detachment of the organic solvent and hydration) were monitored over time by means of UV-Vis spectroscopy (see Figure 4 in the Results section). As these studies revealed rather slow re-equilibration (roughly 5 h were needed to complete the process) and progressive aggregation/precipitation of the hydrated compound, the samples used for the titration experiments were kept under continuous stirring for 72 h by means of a mechanic stirrer prior to acquisition of the fluorescence spectra. While preparing the solutions, the DNA concentration in each sample was measured by acquisition of the UV-spectrum prior to ligand addition. Namely, the tabulated molar extinction coefficient value ε(260 nm) = 13,200 M^−1^cm^−1^ was used to derive the molarity in base pairs [58]. After this measurement, a nominal volume of 20 μL of ligand stock in either DMSO or DMF was added to 3 mL of DNA solution in PBS by means of a micro-pipette (Brand). The absorption spectrum in the 300–500 nm spectral region was acquired readily after, when re-equilibration was still negligible. The absorption at 395 nm was then used to perform ex-post correction of the fluorescence data with respect to fluctuations in the ligand concentration owing to the pipette limited precision (≈2% according to the supplier).

### 3.2. Spectroscopy Experiments

IR spectra were acquired by the ATR method with a Nicolet iS10 instrument, over the range 4000–600 cm^−1^. FTIR measurements in external reflection mode (FTIR-ER) were carried out with an Alpha Bruker FTIR portable spectrophotometer, equipped with a DTGS detector. Spectra were collected between 800 cm^−1^ and 375 cm^−1^, with 4 cm^−1^ resolution and 200 scans. The background was periodically acquired using a flat gold mirror. In the following, intensities are denoted as: vs = very strong, s = strong, m = medium, w = weak and vw = very weak. ^1^H and ^13^C(APT) solution NMR spectra were recorded on a Bruker Avance I 400 MHz spectrometer. ^1^H and ^13^C data are reported as follows: chemical shifts (in ppm and referenced to internal TMS), integration, multiplicity (s = singlet, d = doublet, t = triplet, q = quartet, m = multiplet), and coupling constants (in Hz). Deuterated solvent for NMR measurements, CD_2_Cl_2_, was used as received from Eurisotope. Fluorescence spectra were acquired on a PTI Fluorescence Master System fluorimeter. The spectra were automatically corrected with respect to the lamp spectral radiance and detector quantum efficiency by exploiting a feature of the acquisition software, Felix 2000. The values of fluorescence quantum yield were assessed by comparison to a tabulated standard, namely dimethyl-POPOP dissolved in cyclohexane, Φ_fluo_ = 0.95 [65], and were corrected with respect to solvent refractive index. HRMS spectra were recorded on MALDI Tof-Tof Autoflex III instrument. Elemental composition analyses were performed with a Perkin Elmer CHN Analyser 2400 Series II instrument. TGA and DSC were performed simultaneously with a Netzsch STA 409 PC Luxx instrument (temp. range RT-600 °C, rate of 10 °C min^−1^, N_2_ atmosphere). Gas chromatography-mass spectrometry. UV-Vis absorption spectra were recorded by means of a Perkin Elmer Lambda 2 spectrophotometer. 

### 3.3. Powder X-ray Diffraction

Powder X-ray diffraction qualitative measurements were carried out in the 3–35° 2θ range with steps of 0.02° and time per step of 1 s using a Bruker AXS D8 Advance vertical-scan θ:θ diffractometer, equipped with a sealed X-ray tube (Cu Kα, λ = 1.5418 Å), a Bruker Lynxeye linear position-sensitive detector, a Ni filter in the diffracted beam and the following optical components: primary beam Soller slits (2.5°), fixed divergence slit (0.5°), antiscatter slit (8 mm). The generator operated at 40 kV and 40 mA. Powdered samples (~50 mg) of **1** were deposited in the cavity of an aluminum sample-holder 0.2 mm deep. All the samples used for the spectroscopic characterization were preliminary checked by elemental analysis and PXRD.

### 3.4. Synthesis of 1-methyl-5-methoxyindole

This compound was synthetized according to the procedure reported in the literature [66].

### 3.5. Synthesis of BMIP

This compound was synthetized according to the procedure reported in the literature [38].

### 3.6. Synthesis of [Zn_2_(µ-O)_2_(BMIP)_2_]

In a Schlenk tube BMIP (100 mg, 0.257 mmol, 1.1 eq.) was mixed with 2-methoxyethanol (5 mL) and heated to 90 °C to allow complete dissolution while stirring. Zn(ClO_4_)_2_·6H_2_O (86.9 mg, 0.233 mmol, 1 eq.) was added and the solution turned to a transparent dark red color. Triethylamine (1 mL) was slowly added dropwise: modest precipitation of a dark yellow solid was noted. The reaction mixture was then vigorously stirred and refluxed for 6 h, then slowly allowed to cool to room temperature. The crude product was filtered on a glass filter funnel and subsequently washed with 2-methoxyethanol, distilled water and methanol. The isolated yellow solid was transferred into a vial and dried under vacuum at 120 °C for 3 h.

^1^H-NMR(CD_2_Cl_2_), δ(ppm): 7.98 (diketo, *s*, 4H); 7.73 (diketo, *d*, 4H, 4J = 2.35 Hz); 7.70 (enol, s, 4H); 7.68 (enol, *d*, 4H, 4J = 2.34 Hz); 7.22 (enol, *d*, 4H, 3J = 8.84 Hz); 7.16 (diketo, d, 4H, 3J = 8.85 Hz); 6.88 (enol, d, 4H, 4J = 2.34 Hz, 3J = 8.85 Hz); 6.82 (diketo, d, 4H, 4J = 2.34 Hz, 3J = 8.85 Hz); 6.40 (enol, s, 2H); 4.19 (diketo, s, 4H); 3.83 (enol, s, 12H), 3.76 (diketo, s, 12H); 3.75 (diketo, s, 12H). ^13^C-NMR(CD_2_Cl_2_), δ(ppm): 188.0 (CO); 156.6 (C); 138.1 (CH); 132.9 (C), 132.5 (C); 127.3 (C); 113.4 (CH); 110.6 (CH); 10.6 (CH); 55.6 (OCH_3_); 55.3 (CH_2_); 33.7 (NCH_3_). IR (ATR) ṽ(cm^−1^): 3010 (vw); 2927 (vw); 2832 (vw); 1580 (w); 1520 (vs); 1480 v(s); 1407, (s); 1379 (s); 1362 (s); 1290 (m); 1267 (m); 1212 (vs); 1142 (m); 1097 (vs);974 (m); 896 (m); 859(m); 801 (s); 773 (vs); 695 (vs); 507 (m). HMRS-(TOF-ESI): *m*/*z* calcd. for C_46_H_44_N_4_O_10_Zn_2_Na: 966.6696 [M + Na]^+^; found: 966.6675 [M + Na]^+^. Elem. Anal. Calcd. for C_46_H_44_N_4_O_10_Zn_2_ (FW = 943.68 g/mol): C = 58.55%, H = 4.70%, N = 5.94%; found: C = 59.18%, H = 4.95%, N = 6.24%.

### 3.7. Synthesis of [Zn_2_(µ-O)_2_(BMIP)_2_(DMSO)]—Direct Method

In a Schlenk tube, BMIP (101.5 mg, 0.26 mmol, 1.1 eq.) was mixed with DMSO (2 mL) and heated to 40 °C to allow complete dissolution while stirring. Zn(ClO_4_)_2_·6H_2_O (88.07 mg, 0.236 mmol, 1 eq.) was added and the solution turned to a transparent dark orange color. Triethylamine (1 mL) was slowly added dropwise: modest precipitation of a light-yellow solid was noted. The reaction mixture was then vigorously stirred and refluxed for 4 h, then slowly allowed to cool to room temperature. The crude product was filtered on a glass filter funnel and subsequently washed with DMSO, distilled water and methanol in the end. The isolated yellow solid was transferred into a vial and dried under vacuum at 90 °C for 3 h.

### 3.8. Synthesis of [Zn_2_(µ-O)_2_(BMIP)_2_(DMSO)]—Indirect Method

In a 10 mL vial [Zn_2_(µ-O)_2_(BMIP)_2_] (20 mg, 0.02 mmol) was dissolved in DMSO (1.0 mL) and stirred for 12 h at room temperature. A yellow precipitate formed: it was filtered on a small Hirsch filter funnel and washed with diethyl ether (5 × 5 mL). The isolated yellow solid was transferred into a vial and dried under vacuum at 90 °C for 3 h.

^1^H-NMR(CD_2_Cl_2_), δ(ppm): 8.00 (diketo, s, 4H); 7.85 (diketo, d, 4H, ^5^J = 2.35 Hz); 7.81 (enol, s, 4H); 7.80 (enol, d, 4H, ^5^J = 2.54 Hz); 7.33 (enol, d, 4H, ^3^J = 8.90 Hz); 7.27 (diketo, d, 4H, ^3^J = 8.85 Hz); 6.99 (enol, dd, 4H, ^5^J = 2.34 Hz, ^3^J = 8.85 Hz); 6.94 (diketo, dd, 4H, ^5^J = 2.34 Hz, ^3^J = 8.85 Hz); 6.69 (enol, s, 2H); 4.31 (diketo, s, 4H); 3.87 (enol, s, 12H), 3.85 (diketo, s, 12H); 2.58 (DMSO, s, 6H). IR (ATR) ṽ(cm^−1^): 3010 (vw); 2927 (vw); 2832 (vw); 1580 (w); 1520 (vs); 1475 v(s); 1408, (s); 1382 (s); 1365 (vs); 1293 (s); 1267 (s); 1208 (vs); 1126 (vs); 1108 (s); 1095 (vs); 1131 (m); 1008 (m); 970 (m); 963 (m); 894 (m); 860 (m); 808 (vs); 692 (vs); 510 (m); 434 (m). Elem. Anal. Calcd. for C_48_H_50_N_4_O_11_Zn_2_ (FW = 1021.81 g/mol): C =56.42%, H = 4.93%, N = 5.48%; found: C = 56.05%, H = 5.01%, N = 5.84%. The IR spectrum is reported in Supplementary Materials Figure S13.

### 3.9. Synthesis of [Zn_2_(µ-O)_2_(BMIP)_2_(NMI)]

In a 10 mL vial [Zn_2_(µ-O)_2_(BMIP)_2_] (20 mg, 0.02 mmol) was dissolved in 1-methylimidazole (1.0 mL) and stirred for 12 h at room temperature. A yellow precipitate formed: it was filtered on a small Hirsch filter funnel and washed with diethyl ether (5 × 5 mL). The isolated yellow solid was transferred into a vial and dried under vacuum at 100 °C for 4 h.

### 3.10. Synthesis of [Zn_2_(µ-O)_2_(BMIP)_2_(NMI)]—Ligand Exchange

In a 10 mL Schlenk tube [Zn_2_(µ-O)_2_(BMIP)_2_(DMSO)] (51.7 mg, 0.05 mmol) was dissolved in CH_2_Cl_2_ (4 mL) and then 1-methylimidazole (400 µL) was added. The solution was stirred for 12 hrs at room temperature. A yellow precipitate formed: it was filtered on a small Hirsch filter funnel and washed with diethyl ether (5 × 5 mL). The isolated yellow solid was transferred into a vial and dried under vacuum at 100 °C for 4 h.

^1^H-NMR(CD_2_Cl_2_), δ(ppm): 8.10 (diketo, s, 4H); 7.85 (diketo, d, 4H, ^5^J = 2.35 Hz); 7.81 (enol, s, 4H); 7.80 (enol, d, 4H, ^5^J = 2.54 Hz); 7.56 (NMI, sb, 1H); 7.34 (enol, d, 4H, ^3^J = 8.88 Hz); 7.28 (diketo, d, 4H, ^3^J = 8.89 Hz); 7.07 (NMI, sb, 1H); 6.99 (enol, dd, 4H, ^5^J = 2.50 Hz, ^3^J = 8.89 Hz); 6.95 (NMI, sb, 1H); 6.94 (diketo, dd, 4H, ^5^J = 2.35 Hz, ^3^J = 8.88 Hz); 6.51 (enol, s, 2H); 4.31 (diketo, s, 4H); 3.95 (enol, s, 12H); 3.88 (enol, s, 12H); 3.87 (enol, s, 24H), 3.71 (NMI, s, 3H). IR (ATR) ṽ(cm^−1^): 2992 (vw); 2932 (w); 2829 (w); 1580 (w); 1520 (vs); 1474 (vs); 1422, (m); 1418 (m); 1408 (s); 1385 (m); 1364 (s); 1211 (vs); 1095 (s); 1034 (m); 973 (m); 1131 (m); 948 (w); 842 (w); 771 (s); 694 (s); 860 (m); 518 (m). Elem. Anal. Calcd. for C_50_H_50_N_6_O_10_Zn_2_ (FW = 1025.78 g/mol): C = 58.54%, H = 4.91%, N = 8.19%; found: C = 59.01%, H = 4.95%, N = 8.30%. The IR spectrum is reported in Supplementary Materials Figure S14.

## 4. Conclusions

[Zn_2_(µ-O)_2_(BMIP)_2_] (BMIP = 1,3-bis(5-methoxy-1-methyl-1*H*-indol-3-yl)propane-1,3-dione), **1**, a novel, highly stable zinc(II) µ-oxo-bridged dimeric complex, was synthesized in quantitative yield according to original procedures. Its complex keto-enolic tautomerism was elucidated by combining NMR, IR and UV-Vis spectroscopy. The ability of the zinc(II) ion to change coordination number in the presence of highly electrophilic moieties when caged within the ligand dimer was unravelled, and the coordination with the organic solvent DMSO and with the base scaffold NMI was studied in detail. Finally, high affinity of the compound for DNA in pseudo-physiologic environment was demonstrated by means of fluorescence spectroscopy. The chemico-physical parameters characterizing the binding were estimated by fitting the binding isotherms according to the independent binding site model. In conclusion, **1** exhibits the essential properties to be considered as a starting scaffold compound for the development of a novel family of Zn-based DNA-targeting metallodrugs. Further efforts should be devoted to improve water solubility of these drug substances, which is actually very poor, in order to increase their bioavailability. Presently, attempts in this sense are being undertaken in our laboratories. Namely, we are adopting the strategy of modifying the substitutions on the nitrogen atoms of the indole rings of the ligand inserting polar groups. Other modifications will be explored on the benzene rings.

## Figures and Tables

**Table 1:** IR frequencies (cm^−1^) of BMIP, **1**, **2** and **3**.

Compound	ν_C–H_	ν_C–H_	ν_C=O_	ν_C–O–C_	ν_C=N_ NMI	ν_C–N_ NMI	Ν_ρ(Me)_ DMSO	ν_(S=O)_ DMSO	ν_Zn–O_	ν_Zn–O_ DMSO
**ligand**		2939	1620	1210						
**1**	3020	2950	1520	1260					507	
**2**	3020	2950	1520	1260			1031	961	510	435
**3**	3020	2950	1520	1260	1421	1285			518	

**Table 2:** UV-Vis absorption properties of **1** in different solvents: peak wavelength of the first absorption band, λ_abs,1_, and of the second absorption band, λ_abs,2_; relative intensity of the first absorption peak, f_1_; fraction of diketo tautomers according to NMR, f_Diketo,NMR_; average molar extinction coefficient, <ε> (see text for details).

Solvent	λ_abs,1_ (nm)	λ_abs,2_ (nm)	f_1_	f_Diketo,NMR_	<ε> (M^−1^cm^−1^)
DCM	313 (35,000)	392 (32,900)	0.79	0.85	34,600
DMF	302	392	0.57		44,100
DMSO	309 (41,500)	393 (43,000)	0.45	0.46	42,400
EtOH	311	397	0.76		--

**Table 3:** Fluorescence properties of **1** in selected solvents: peak fluorescence emission wavelengths upon excitation at the enol, λ_emi,enol_, and diketo, λ_emi,diketo_, absorption peak; peak fluorescence excitation wavelengths with fluorescence recording at the enol emission peak, λ_exc,enol_, and at 350 nm, λ_exc,diketo_; fluorescence quantum yield of the enol tautomers, Φ_fluo,enol_.

Solvent	λ_emi,enol_ (nm)	λ_emi,diketo_ (nm) ^†^	λ_exc,enol_ (nm)	λ_exc,diketo_ (nm)	Φ_fluo,enol_
DCM	426	403	326 (398)	315	0.06
DMF	424	405	400 (325)	280 (309)	0.08
DMSO	428	403	400, 408 (322)	293, 304	0.12
EtOH	447	413	401 (325)	292	0.20

^†^ reconstructed by subtraction of the out-of-peak excited fluorescence of the enol tautomers, see text for detailed procedure.

**Table 4:** Fitting parameters obtained by interpolation of the experimental binding isotherms of Figure 6 with the independent binding site model, Equation (1).

Solvent	K_diss_ (M)	*n* (ligands/bp)	F_0_ (a.u.)	δF (μM^−1^)
DMSO	0.69 ± 0.32	0.26 ± 0.05	2.07 ± 0.03	−0.098 ± 0.004
DMF	0.87 ± 0.39	0.29 ± 0.05	1.19 ± 0.02	−0.057 ± 0.005

## Data Availability

Data is contained within the article and supplementary material.

## Supplementary Materials

The following materials are available online at https://www.mdpi.com/article/10.3390/ph14080760/s1, Figure S1: ^1^H-NMR spectrum of **1**, Figure S2: ^13^C-NMR spectrum of **1**, Figure S3: IR spectrum of **1**, Figure S4: Fluorescence excitation spectra of **1** in selected solvents, Figure S5: Reconstructed fluorescence emission spectra of the diketo tautomers of **1** in selected solvents, Figure S6: Absorption and fluorescence spectra of **1** in water and bound to DNA, Figure S7: PRXD pattern, Figure S8: ^1^H-NMR spectrum of **2**, Figure S9: DSC/TGA trace of **2**, Figure S10: DSC/TGA trace of **1**, Figure S11: ^1^H-NMR spectrum of **3**, Figure S12: DSC/TGA trace of **3**, Figure S13: IR spectrum of **2**, Figure S14: IR spectrum of **3**.
