# Supplementary material for: Synthesis, Characterization and DNA-Binding Affinity of a New Zinc(II) Bis(5-methoxy-indol-3-yl)propane-1,3-dione Complex
Source: Pharmaceuticals (Basel). 2021 Aug 3;14(8):760. doi: 10.3390/ph14080760 (PMC8398859; doi:10.3390/ph14080760)
Supplement: Supplementary file 1 [file pharmaceuticals-14-00760-s001.zip › pharmaceuticals-1309675-supplementary.pdf]

pharmaceuticals

Article

Synthesis, Characterization and DNA-Binding Affinity of a New Zinc(II) Bis(5-methoxy-indol-3-yl)propane-1,3-dione Complex

Luca Scapinello [1], Guglielmo Vesco [1], Luca Nardo [1], Angelo Maspero[1,*], Federico Vavassori [1], Simona Galli [1], Andrea Penoni [1]

1 Università degli Studi dell'Insubria – Dept. of Science and High Technology, Via Valleggio 11, 22100 Como (CO); luca.scapinello@uninsubria.it (L.S.), gvesco@uninsubria.it (G.V.); luca.nardo@uninsubria.it (L.N.); angelo.maspero@uninsubria.it (A.M.); f.vavassori1@uninsubria.it (F.V.); simona.galli@uninsubria.it (S.G.); andrea.penoni@uninsubria.it (A.P.).

* Correspondence: angelo.maspero@uninsubria.it ; Tel.: +39 031 238 6472

Supplementary Online Materials

Citation: Scapinello, L.; Vesco, G.; Nardo, L.; Maspero, A.; Vavassori, F.; Galli , S. Synthesis, Characterization and DNA-Binding Affinity of a New Zinc(II) Bis(5-methoxy-indol-3-yl) propane-1,3-dione Complex. *Pharmaceuticals* **2021**, *14*, 760. https://doi.org/10.3390/ph14080760

Academic Editor: Jean Jacques Vanden Eynde

Received: 6 July 2021
Accepted: 28 July 2021
Published: 3 August 2021

Publisher's Note: MDPI stays neutral with regard to jurisdictional claims in published maps and institutional affiliations.

Copyright: © 2021 by the authors. Submitted for possible open access publication under the terms and conditions of the Creative Commons Attribution (CC BY) license (http://creativecommons.org/licenses/by/4.0/).

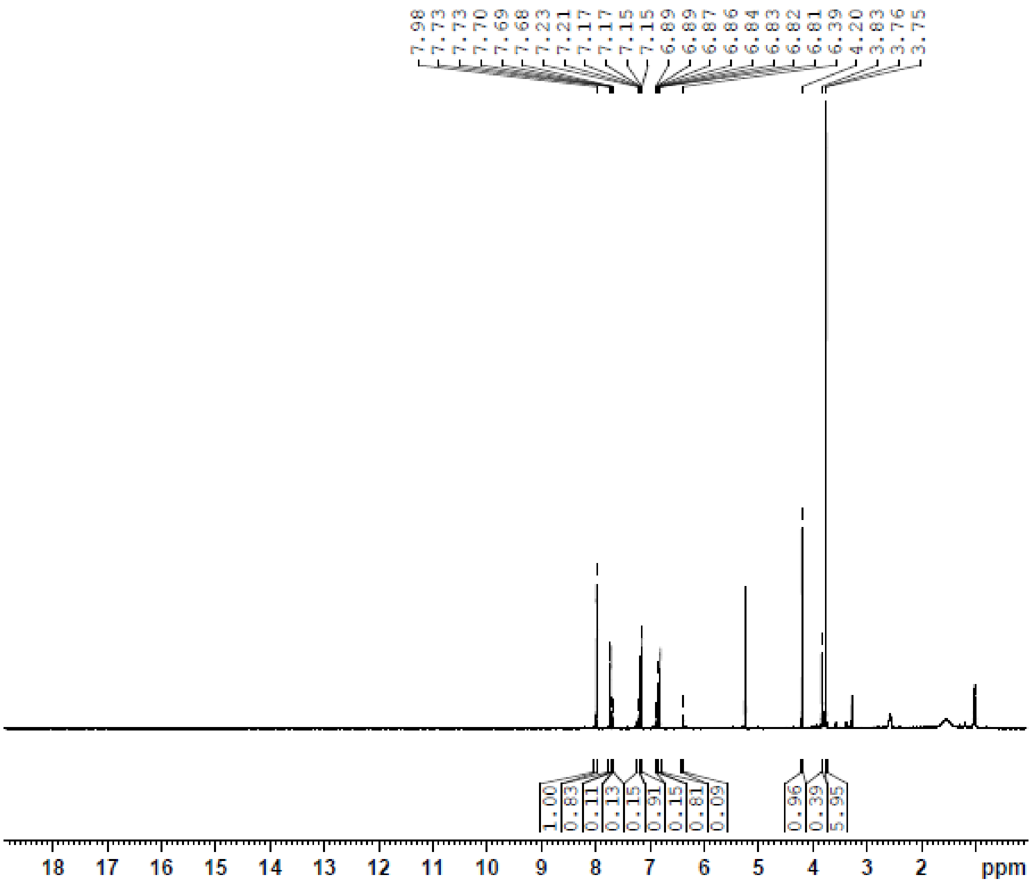

Figure S1. ^{1}H-NMR spectrum of **1**.

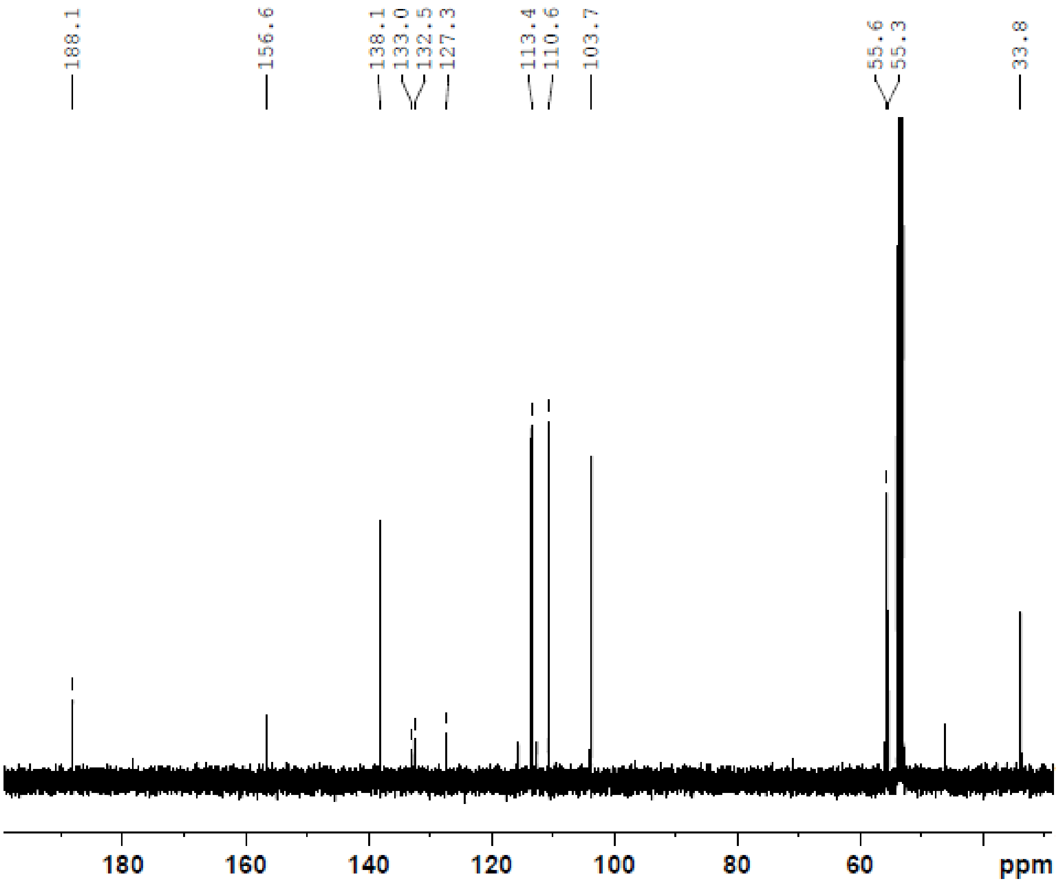

Figure S2. ^{13}C-NMR spectrum of **1**.

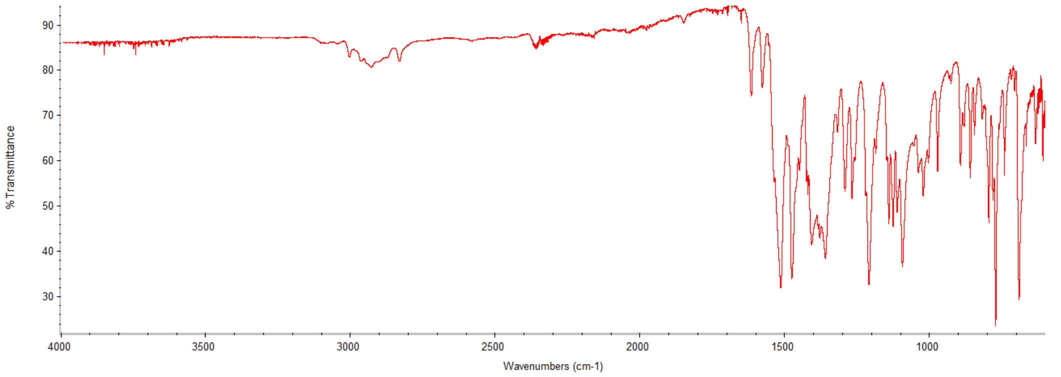

Figure S3. IR absorption spectrum of **1** in the 600 cm^{-1} – 4000 cm^{-1} spectral region.

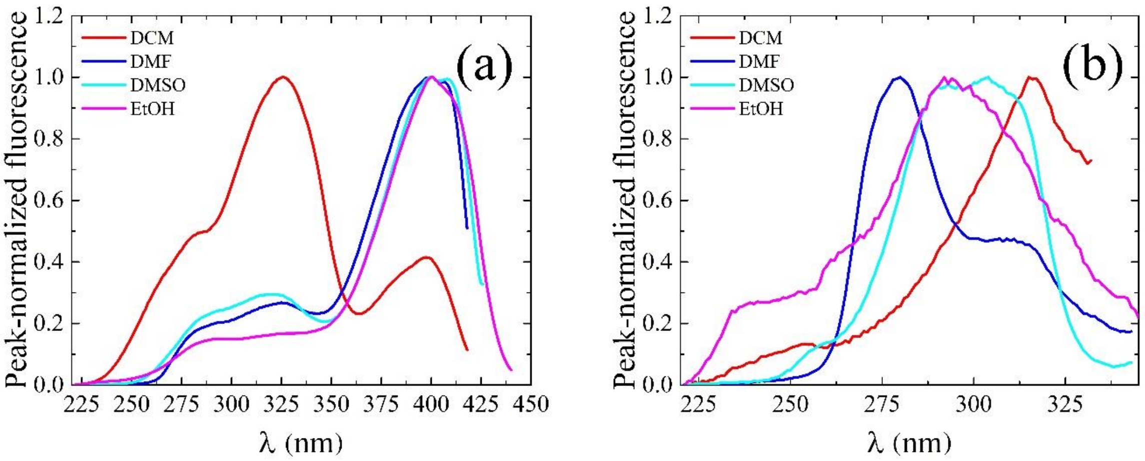

Figure S4. Fluorescence excitation spectral line-shapes of **1** in different solvents with fluorescence detection at a) the enol emission peak and b) 350 nm (in the region of the diketo-emission shoulder).

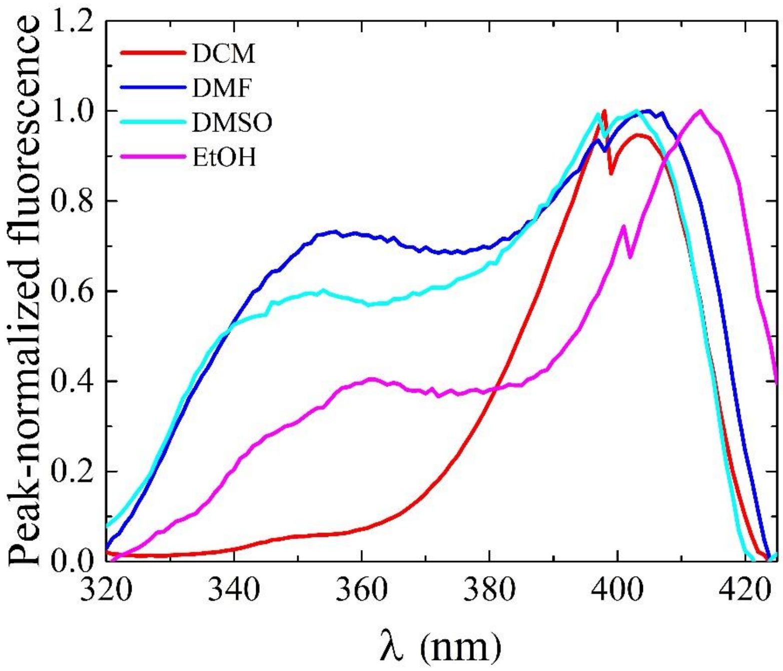

Figure S5. Reconstructed emission spectra of the diketo tautomers of **1**. With the exception of dichloromethane, the spectra exhibit two well separated peaks. This might indicate the coexistence of cis- and trans-diketo tautomers in polar environments.

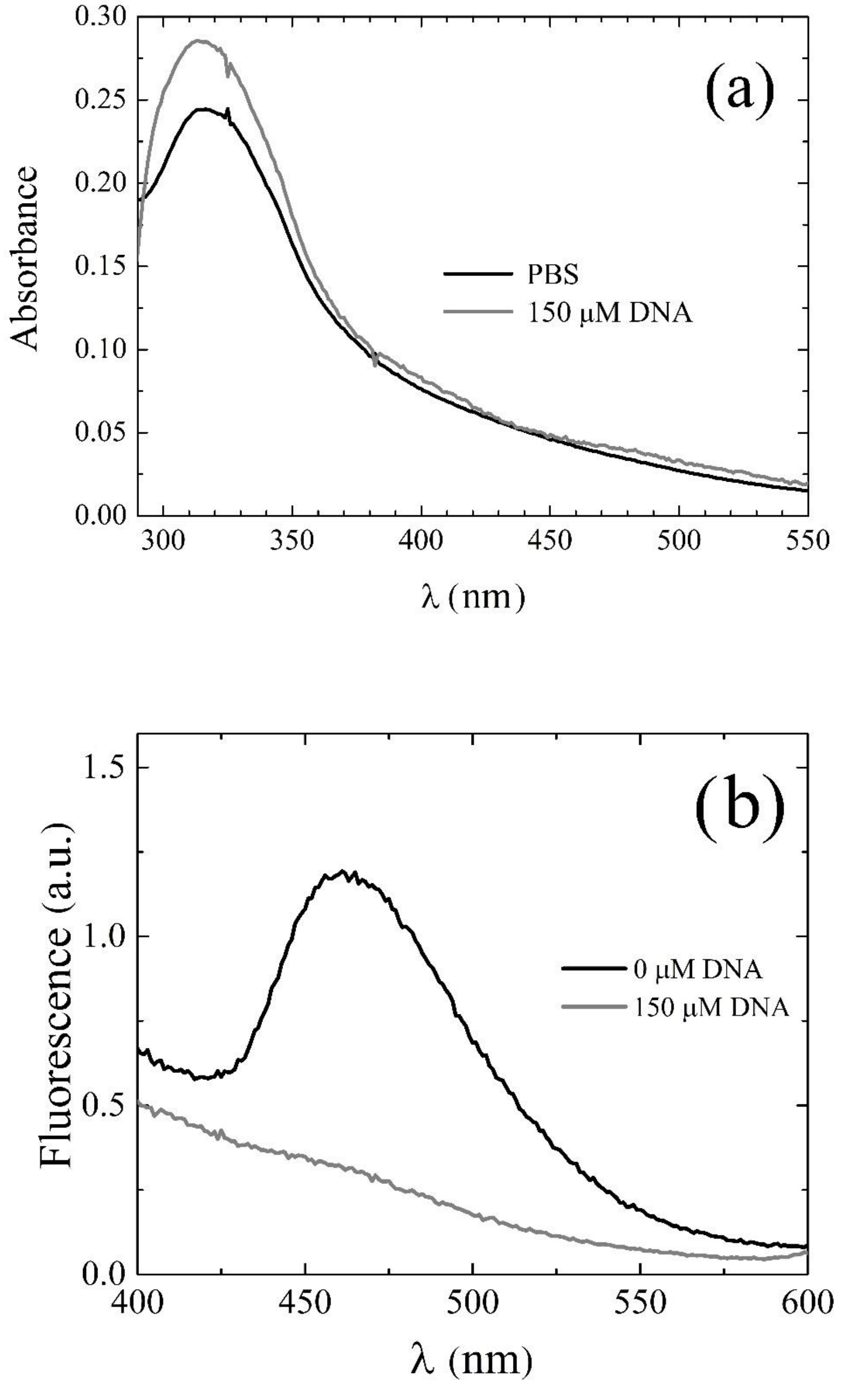

Figure S6. a) Absorption and b) emission spectra of **1** in aqueous environment (PBS) in the absence (black line) and presence (gray line) of DNA. The compound was preliminarily solubilized in DMF. Fluorescence was elicited upon excitation at 320 nm.

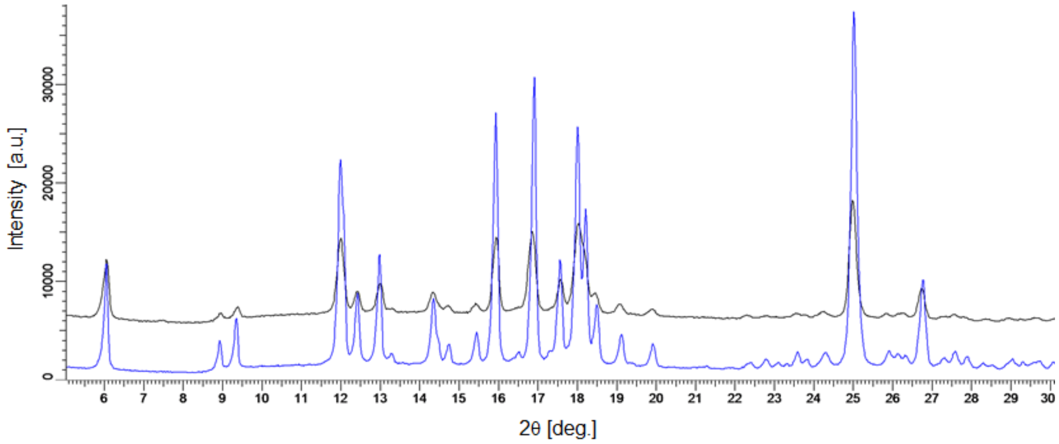

Figure S7. Low-to-medium angle portion of the powder X-ray diffraction pattern of two batches of complex **1** at comparison.

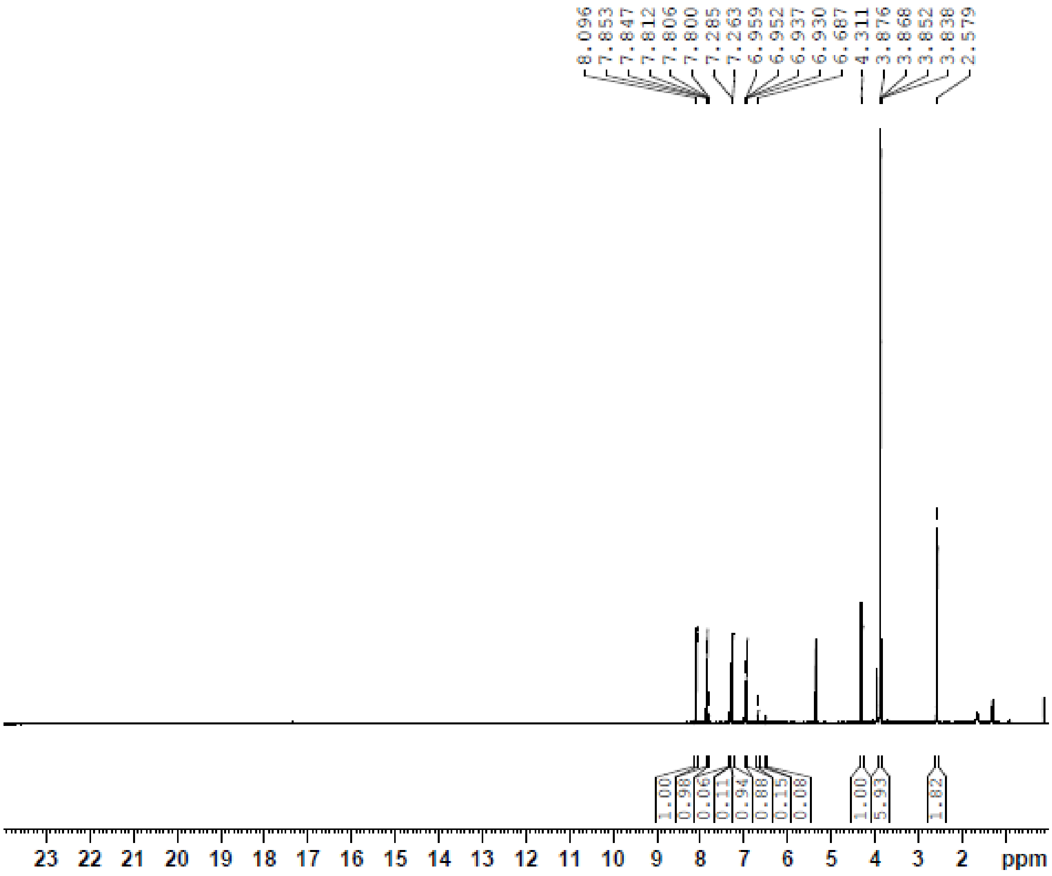

Figure S8. ^{1}H-NMR spectrum of **2**.

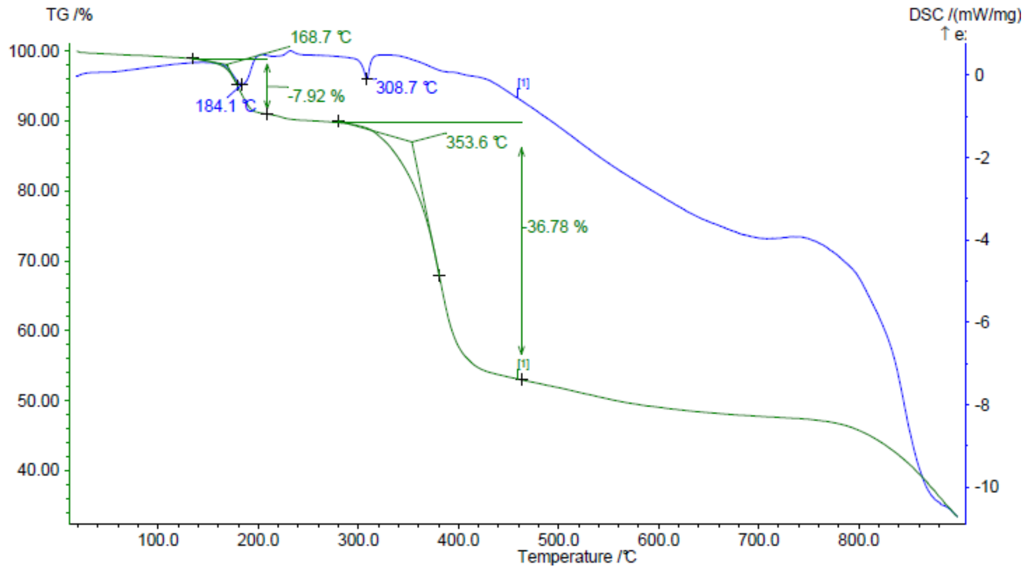

Figure S9. DSC (blue line) and TGA (green) traces of **2**.

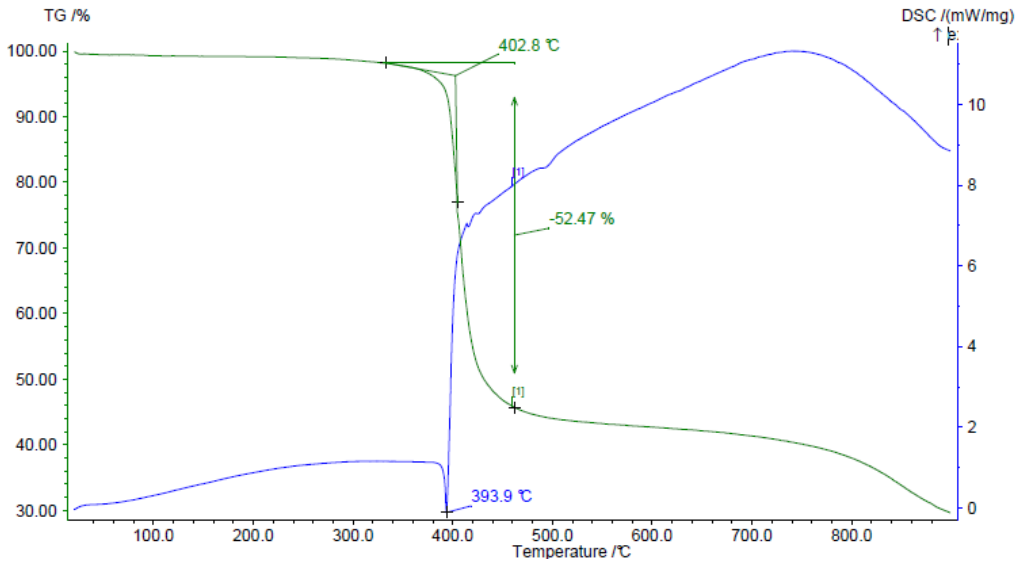

Figure S10. DSC (blue line) and TGA (green) traces of **1**.

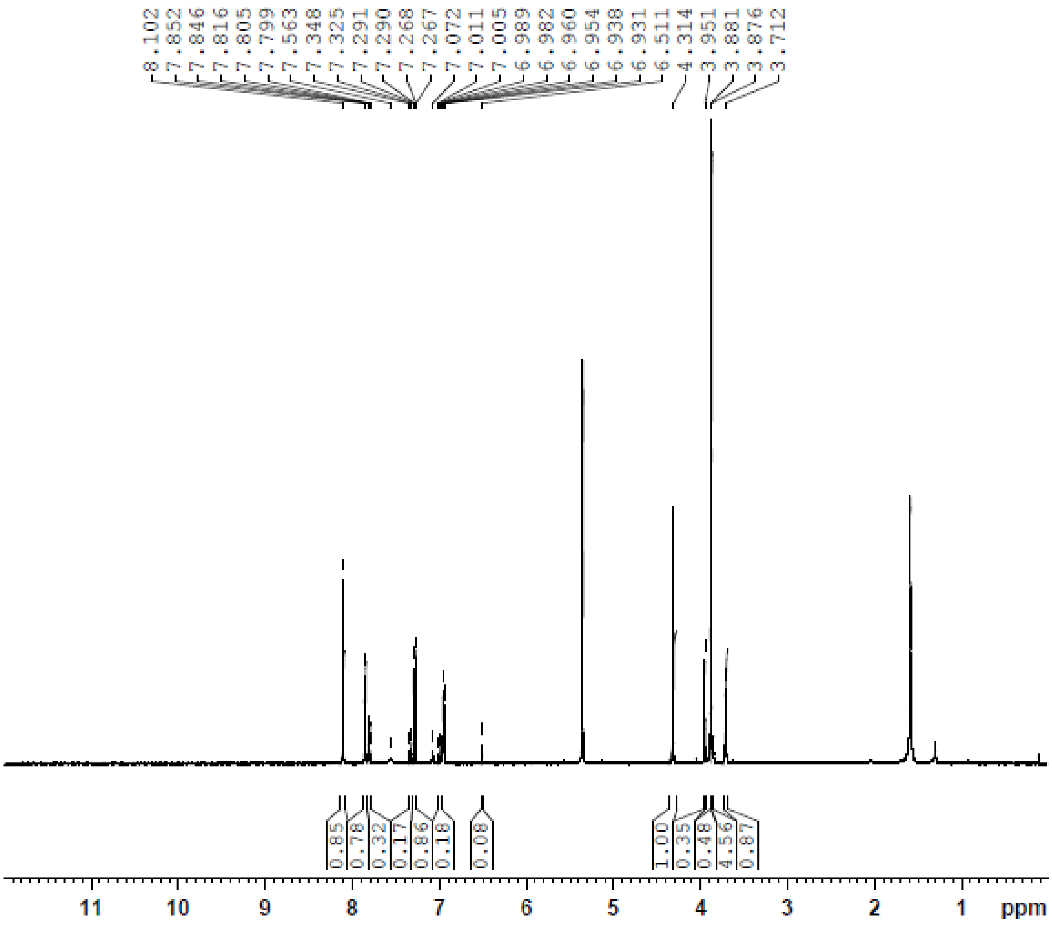

Figure S11. ^{1}H-NMR spectrum of **3**.

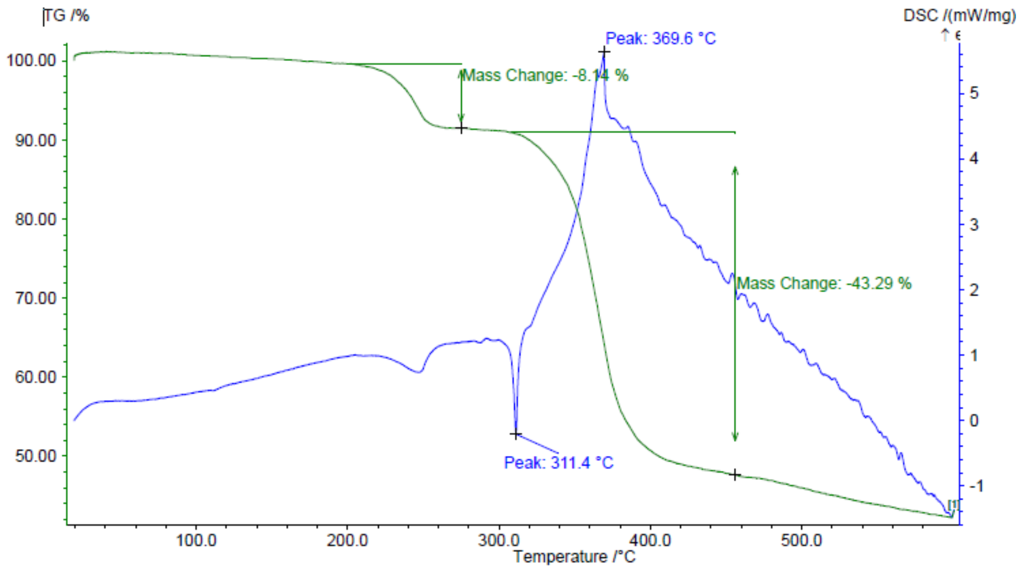

Figure S12. DSC (blue line) and TGA (green) traces of **3**.

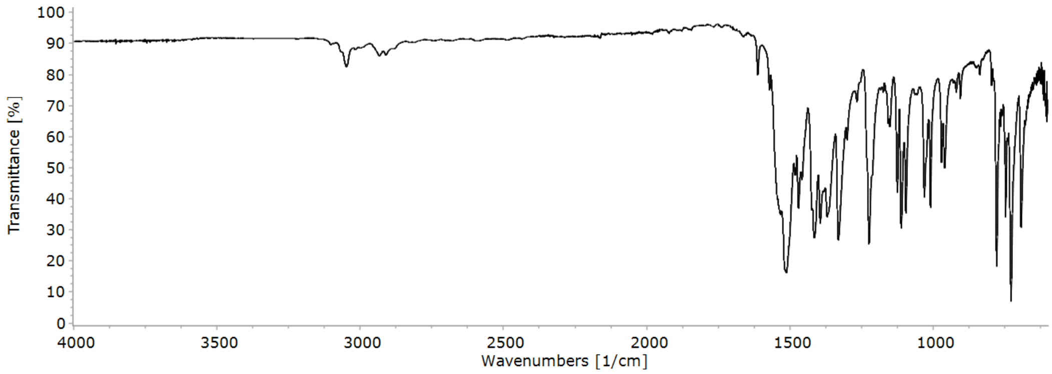

Figure S13. IR absorption spectrum of **2** in the 600 cm^{-1} – 4000 cm^{-1} spectral region.

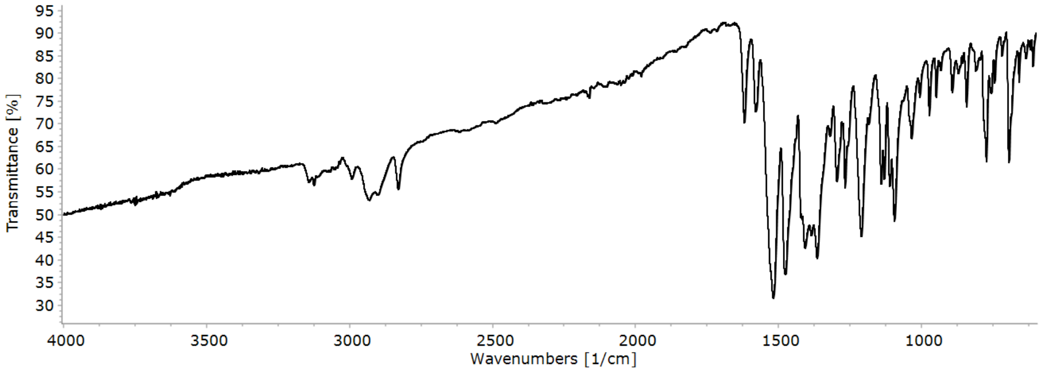

Figure S14. IR absorption spectrum of **3** in the 600 cm^{-1} – 4000 cm^{-1} spectral region.
